# Quantitative [^68^Ga]Ga-PSMA-11 PET biomarkers for the analysis of lesion-level progression in biochemically recurrent prostate cancer: a multicentre study

**DOI:** 10.1038/s41598-023-45106-2

**Published:** 2023-10-17

**Authors:** Jake Kendrick, Roslyn J. Francis, Ghulam Mubashar Hassan, Pejman Rowshanfarzad, Jeremy S. L. Ong, Nathaniel Barry, Branimir Rusanov, Martin A. Ebert

**Affiliations:** 1 School of Physics, Mathematics and Computing, The University of Western Australia, Perth, WA Australia; 2 Centre for Advanced Technologies in Cancer Research, Perth, WA Australia; 3 Medical School, University of Western Australia, Crawley, WA Australia; 4 Department of Nuclear Medicine, Sir Charles Gairdner Hospital, Perth, WA Australia; 5 Australian Centre for Quantitative Imaging, University of Western Australia, Crawley, WA Australia; 6 Department of Nuclear Medicine, Fiona Stanley Hospital, Murdoch, WA Australia; 7 Department of Radiation Oncology, Sir Charles Gairdner Hospital, Perth, WA Australia; 8 5D Clinics, Claremont, WA Australia

**Keywords:** Tumour biomarkers, Urological cancer

## Abstract

[^68^Ga]Ga-PSMA-11 PET has become the standard imaging modality for biochemically recurrent (BCR) prostate cancer (PCa). However, its prognostic value in assessing response at this stage remains uncertain. The study aimed to assess the prognostic significance of radiographic patient-level patterns of progression derived from lesion-level biomarker quantitation in metastatic disease sites. A total of 138 BCR PCa patients with both baseline and follow-up [^68^Ga]Ga-PSMA-11 PET scans were included in this analysis. Tumour response was quantified at the lesion level using commonly used quantitative parameters (SUV_mean_, SUV_max_, SUV_peak_, volume), and patients were classified as systemic, mixed, or no-progression based on these response classifications. A total of 328 matched lesions between baseline and follow-up scans were analysed. The results showed that systemic progressors had a significantly higher risk of death than patients with no progression with SUV_mean_ demonstrating the highest prognostic value (HR = 5.70, 95% CI = 2.63–12.37, *p* < 0.001, C-Index = 0.69). Moreover, progressive disease as measured by SUV_mean_ using the radiographic PSMA PET Progression Criteria (rPPP) was found to be significantly prognostic for patient overall survival (HR = 3.67, 95% CI = 1.82–7.39, *p* < 0.001, C-Index = 0.65). This work provides important evidence supporting the prognostic utility of PSMA response quantitation in the BCR setting.

## Introduction

Prostate cancer (PCa) is a malignancy that poses a large burden to global public health with significant patient mortality and morbidity^1^. Localised PCa disease can often be treated curatively, with corresponding good patient outcomes. However, biochemical disease recurrence is common, with between 20 and 40% of patients presenting with rising PSA levels following localised disease treatment that can portend the future development of metastatic disease that is associated with a very poor patient prognosis^2–4^.

Radiographic assessment at the stage of biochemically recurrent (BCR) PCa disease has been transformed by the advent of prostate-specific membrane antigen (PSMA) targeting radioligands, with recent years seeing the development of numerous ligands that bind with the PSMA transmembrane protein to facilitate either diagnostic or therapeutic applications^5–7^. In the BCR PCa stage, PSMA positron emission tomography (PET) imaging has shown superior diagnostic capabilities compared to conventional imaging modalities, such as bone scintigraphy and computed tomography (CT) scanning^8–10^.

PSMA PET/CT imaging enables a non-invasive radiographic assessment of disease progression. Disease progression is often assessed at the patient level, and numerous patient-level criteria have been developed for assessing radiographic progression, including: the Response Evaluation Criteria in Solid Tumors (RECIST 1.1), the PET Evaluation Response Criteria in Solid Tumors (PERCIST), the updated Prostate Cancer Working Group 3 (PCWG3) criteria, and, more recently, PSMA PET-specific response criteria such as the PSMA PET Progression Criteria (PPP) and the Response Evaluation Criteria in PSMA PET/CT (RECIP 1.0)^11–15^. These criteria help standardise the assessment of disease progression and facilitate consistent interpretation of radiographic findings in PSMA PET/CT scans.

While many of these patient-level progression frameworks have demonstrated prognostic utility, particularly the PSMA PET-specific criteria such as the PPP and RECIP 1.0^16,17^, these patient-level classifications might obscure heterogeneity in response at the lesion-level with potentially important clinical implications. There is growing recognition that diversity in molecular profiles among different metastatic sites can manifest in radiographic “mixed response” scenarios, where some lesions exhibit response to administered treatments while others are progressing in either size or uptake, or new disease sites are appearing^18–21^. Furthermore, the prognostic value of PSMA PET quantitation in the response assessment setting has been evaluated primarily in widespread metastatic PCa patients, often undergoing ^177^Lu-PSMA radioligand therapy^16,17,20,22–24^. The prognostic value of PSMA PET response quantitation at early disease stages in patients with more limited metastatic spread remains to be determined.

Comprehensive radiographic lesion-level assessment is complicated by several factors that can influence the progression assessment. There are a suite of possible imaging biomarkers that can be used to assess progression at the lesion level, with some commonly used ones including several standardised uptake value (SUV) measurements such as SUV_max,_ SUV_mean_, SUV_peak_ as well as tumour volume^20,25^. Additionally, the required percentage change in these biomarkers between baseline and follow-up for a lesion to be classified as responding or progressing could greatly impact the treatment response assessment.

This study aimed to: (1) analyse the lesion-level patterns of progression in a cohort of biochemically recurrent (BCR) PCa patients undergoing standard-of-care treatment using several conventionally used imaging biomarkers in PSMA-PET, classifying the response heterogeneity among biomarkers, and (2) determine the correlation between radiographic patient-level patterns of progression, derived from matched lesion-level segmentations, and patient overall survival (OS) in the BCR setting.

## Methods

### Patient cohort

**Figure 1:**
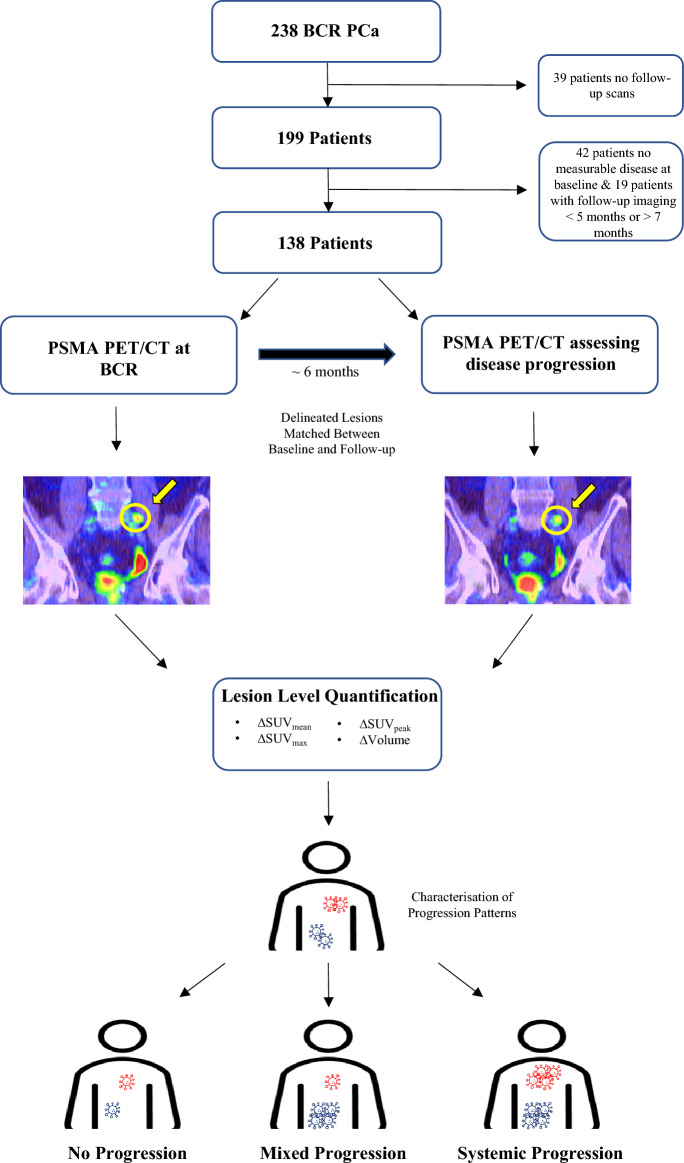
Overview of patient selection and study design.

This retrospective study utilised a cohort of 238 patients with BCR PCa who were imaged as part of a prospective trial registered with the Australian and New Zealand Clinical Trials Registry (ACTRN12615000608561)^8^. Patients were imaged at one of two hospitals in Perth, Western Australia—Sir Charles Gairdner Hospital (SCGH) or Fiona Stanley Hospital (FSH). To be included in this prospective trial, patients had to meet the following criteria: (1) present with biochemically recurrent disease after definitive primary therapy, based on serum prostate-specific antigen (PSA) values, defined as either a PSA level > 0.2 ng/mL at > 6 weeks post radical prostatectomy or a PSA level 2 ng/mL above the previous nadir measurement at 3 months post external beam radiotherapy, and; (2) have either oligometastatic (≤ 3 lesions) or negative disease on conventional staging imaging (abdominopelvic contrast CT and bone scintigraphy). Out of the 238 patients, 199 received both baseline and follow-up [^68^Ga]Ga-PSMA-11 PET/CT scans approximately 6 months later, and therefore, 39 patients were excluded from the analysis. Between the baseline and follow-up scans, patients were treated according to the standard of care as determined by the treating physician, including: active surveillance, radiotherapy to either the prostatic bed or metastatic disease sites, additional surgical procedures, systemic hormonal treatment, or chemotherapy. All methods carried out in this study were conducted in accordance with the ethical standards of the SCGH Human Research Ethics Committee, and appropriate ethics approval was obtained (RGS1736). The research was conducted in line with the principles of the Declaration of Helsinki. An overview of the patient inclusion criteria and study design is provided in Fig. 1.

### Scan acquisition

[^68^Ga]Ga-PSMA-11 PET/CT scans were acquired 60 min after the intravenous injection of 2 MBq/Kg of [^68^Ga]Ga-PSMA-11 on either a Siemens Biograph 64 or a Siemens Biograph 128 PET/CT scanner (CTI Inc, Knoxville TN). Patients were asked to void their bladders just prior to commencement of imaging. PET data was acquired immediately after a low dose CT acquisition (50 mAs, 120 kvP) for attenuation correction, with identical fields of view. PET images were reconstructed to an axial plane pixel size of 4.07 × 4.07 mm^2^, while CT images were reconstructed to a pixel size of either 0.98 × 0.98 mm^2^ or 1.52 × 1.52 mm^2^. Additional scanning protocol information is provided in Supplementary Table 1.

### Manual lesion delineation

An expert nuclear medicine physician (J.O.) retrospectively analysed and segmented PCa lesions in the patient scans. Interpretation of scans was done in accordance with published E-PSMA 5-point scoring guidelines^26^. Areas of elevated tracer uptake were determined to be PCa lesions if they were interpreted as either ‘definitely’ or ‘probably’ positive according to these guidelines. Disease sites were segmented with a semi-automated approach, beginning with a threshold of 3 SUV normalised to the patient body weight (SUV_bw_), applied to the PET image. The segmentation mask generated by this threshold was then manually altered, removing any physiological uptake that was mistakenly included, and adding any pathologic uptake areas that were missed. All segmentations were performed on the MIM Encore software (MIM Software Inc., Cleveland, OH, USA). Lesions were manually matched between baseline and follow-up imaging to enable the quantification of imaging biomarkers at the lesion level. The appearance of new lesions in the follow-up image was noted, and the number of new lesions was quantified.

### Lesion-level biomarker quantification

Lesion-level response was assessed using a variety of different imaging biomarkers quantified for each lesion at both baseline and follow-up imaging. The imaging biomarkers included: SUV_max_, SUV_mean_, SUV_peak_, and volume. Given the non-standardised definition of SUV_peak_, a multitude of different SUV_peak_ definitions were tested. Six different spherical radii were used (ranging from 2.5 to 8.75 mm in increments of 1.25 mm) for two different types of SUV_peak_ definitions (centered on the lesion voxel with the maximum uptake, or iterated over all lesion voxels to determine highest uptake region), yielding 12 different definitions of SUV_peak_ that were investigated (see Supplementary Table 2 for a further description of the biomarkers). The percentage change in biomarkers for each lesion between baseline and follow-up was quantified and used to classify lesions into three response categories—partial response, stable, and progressing. Lesion-level response classifications were determined using three different response assessment thresholds: ± 20%, ± 30%, and ± 40% change in each considered biomarker.

### Patient-level patterns of progression

Using the lesion-level classifications for each biomarker at each different response assessment threshold, patient-level patterns of progression were determined and analysed. These patterns were predefined prior to the conduction of the analysis according to previously published patterns of progression for patients with metastatic disease^21^, in which patients were classified as having: (1) systemic progression, (2) mixed progression, or (3) no progression. These patterns are defined in Table 1.

**Table 1:** Lesion-level patterns of progression defined. Progressing lesions includes lesions that are progressing between baseline and follow-up, as well as new lesions identified at follow-up imaging^21^.

Progression criteria	Patients with ≤ 3 baseline lesions	Patients with > 3 baseline lesions
Systemic	≥ 2 lesions with PD	≥ 3 lesions with PD
Mixed	1 lesion with PD	≤ 2 lesions with PD
No progression	No PD in any lesion	No PD in any lesion

PD, Progressive disease.

A special case of the patient-level patterns described above should be noted. Considering a ± 30% threshold for lesion progression, and combining the two categories of “systemic” and “mixed” progression into a single progression category, “progressive disease” (PD), the criteria elucidated above become almost equivalent to the PPP criteria^14^. The exception is that as per the aims of this study, progression is assessed purely radiographically, without the requirement of confirmatory clinical or laboratory data. We may therefore define the radiographic PPP (rPPP) interpretation, where patient-level PD results from one of the following: (1) Increase in the size or uptake of any lesion by ≥ 30%, or (2) Appearance of 1 or more new PSMA-positive lesions. This definition is consistent with the PSMA PET/CT response assessment consensus statement^27^, where the appearance of even one new lesion in early recurrent disease is to be considered PD.

### Statistical analysis

Comparisons of continuous variables (baseline PSA values and tumour burden) between progression groups were conducted using the Mann–Whitney U-test. Two-way comparisons of the quantified biomarker distributions between baseline and follow-up were performed using the Wilcoxon-signed rank test. Both tests were performed using the SciPy Python package version 1.7.3. The association between patient-level patterns of progression categories and patient OS (measured from the date of classification of progression pattern at follow-up imaging until the date of death) was assessed using the Kaplan–Meier method with the log rank test and univariate Cox regression analysis. Hazard ratios (HRs) were derived, and Harrell's concordance index (C-index) was calculated. Survival analysis was performed with the Lifelines package version 0.27.1. In the case of deriving HRs for all candidate biomarkers at the three thresholds investigated, the Bonferroni correction was applied to correct for multiple testing by multiplying calculated *p* values by the number of tests conducted. In all cases, a *p*-value of less than 0.05 was considered to be a statistically significant difference. All statistical analysis were conducted in Python version 3.9.

### Ethics approval

Ethics approval for undertaking this study was acquired from the Sir Charles Gairdner Hospital Human Research Ethics Committee (RGS1736).

### Consent to participate

Informed consent was obtained from all individual participants included in the study.

## Results

### Patient and lesion characteristics

**Figure 2:**
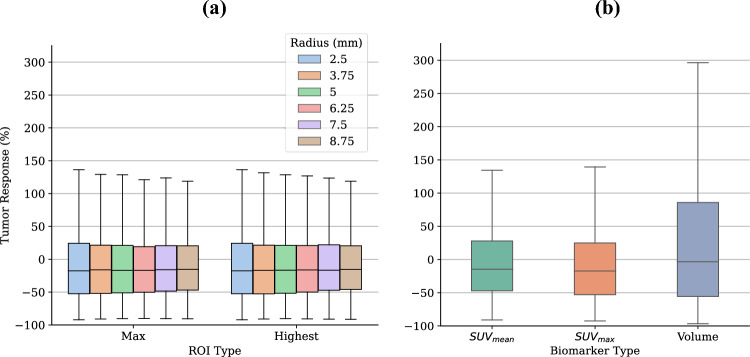
Tumor response percentages, quantified as a percentage change in the respective biomarker between baseline and follow-up imaging, for: (**a**) SUV_peak_ measurements which vary with type (centered on SUV_max_ of the tumor, or the highest uptake region of the tumor) and radius of spherical volume (between 2.5 and 8.75 mm in increments of 1.25 mm), and (**b**) SUV_mean_, SUV_max_, and Volume.

In total, 199 patients with both baseline and follow-up PSMA imaging were included in this study. 42 of these patients (21.1%) had no measurable disease at baseline imaging, resulting in no lesions to match with the follow-up scan. A further 19 patients were excluded for having follow-up imaging greater than 1 month away from the specified 6-month time point due to the potential for this to affect the analysis conducted. From the remaining 138 patients, 536 lesions were identified at baseline imaging, with a median number of 2 lesions identified per patient (range: 1–34). The demographic and clinical characteristics of these patients are presented in Table 2. The majority of patients (n = 75, 54.3%) included in the analysis underwent Androgen Deprivation Therapy (ADT) between scans, 43 (31.2%) underwent radiotherapy, and 5 (3.6%) were administered chemotherapy treatments. Additionally, 39 patients (28.3%) did not receive any treatment between scans and underwent disease surveillance instead. Among the baseline lesions, 208 were not identified at follow-up imaging and were therefore classified as completely responding, leaving a total of 328 matched lesions for which the percentage differences in baseline and follow-up biomarkers could be quantified. Summary statistics of all biomarkers extracted from these lesions at both baseline and follow-up are presented in Table 3, and boxplots of the percentage response according to each biomarker are presented in Fig. 2. A total of 136 new lesions were identified between baseline and follow-up scanning, with 28 patients (20.3%) presenting new disease sites. This was heavily skewed by one particular patient who presented with 46 new disease sites at follow-up imaging. To enable survival analysis, patients were followed up from the time of follow-up scan until either death or date of censoring, with a median follow up time of 67.3 months (range: 6.1–74.3 months).

**Table 2:** Patient characteristics.

Characteristic	All Patients (n = 138)
Age (y)	71 (46–90)
PSA (ng/mL)	3.80 (0.20–42.00)
Gleason score*	
< 8	73
≥ 8	62
Time between baseline and follow-up scan (months)	6.0 (5.3–7.0)
Risk category at baseline^#^	
Low risk	7 (5.1%)
Intermediate risk	60 (43.5%)
High risk	68 (49.3%)
Previous definitive treatment	
Prostatectomy	75
Radiotherapy	63
Administered treatments between imaging	
Active surveillance	39 (28.3%)
ADT	75 (54.3%)
Radiotherapy	43 (31.2%)
Chemotherapy	5 (3.6%)
Number of lesions identified at baseline imaging	2 (1–34)

*Data missing for 3 patients.

^#^Risk categories assigned based on gleason scores and PSA levels at referral. Low risk: PSA < 10 ng/mL and gleason score = 6. Intermediate risk: PSA between 10 and 20 ng/mL or gleason score = 7. High risk: PSA > 20 ng/mL or gleason score > 7. Risk categories not calculated for 3 patients with missing gleason scores.

Continuous data is presented as the median with the range in parentheses, while nominal data is presented as the number with percentage of the whole in parentheses.

PSA, Prostate specific antigen; ADT, Androgen deprivation therapy.

**Table 3:** Differences in quantitative biomarker values between baseline and follow-up lesions.

Biomarker	Spherical Radius (mm)	Baseline		Follow Up		*p* value*
		Median	IQR	Median	IQR	
SUV_peak_ (centered on SUV_max_)	2.50	5.52	3.23–10.86	4.24	2.70–8.04	< 0.001
	3.75	4.72	2.82–8.89	3.64	2.35–6.76	< 0.001
	5.00	4.17	2.51–7.80	3.14	2.13–5.89	< 0.001
	6.25	3.75	2.24–6.95	2.88	2.00–5.30	< 0.001
	7.50	3.35	1.98–6.00	2.52	1.79–4.58	< 0.001
	8.75	2.88	1.70–5.16	2.18	1.55–3.95	< 0.001
SUV_peak_ (highest uptake region)	2.50	5.52	3.23–10.86	4.24	2.70–8.04	< 0.001
	3.75	4.72	2.83–8.97	3.64	2.35–6.80	< 0.001
	5.00	4.20	2.53–7.92	3.19	2.16–5.89	< 0.001
	6.25	3.81	2.30–7.06	2.91	2.02–5.33	< 0.001
	7.50	3.44	2.05–6.16	2.64	1.82–4.67	< 0.001
	8.75	2.95	1.80–5.42	2.25	1.60–4.20	< 0.001
SUV_max_	N/A	5.87	3.45–11.66	4.53	2.78–8.59	< 0.001
SUV_mean_	N/A	4.34	2.72–7.36	3.59	2.20–5.60	< 0.001
Volume (mL)	N/A	0.86	0.36–1.43	0.83	0.33–1.42	0.29

*Wilcoxon-signed rank test.

SUV, Standardised Uptake Value; IQR, Interquartile range.

### Classification discordance

Overall, there was substantial heterogeneity in lesion-level classifications depending on the choice of imaging biomarker and response assessment threshold. When the response assessment threshold was held constant, a considerable number of lesions were classified differently across the range of imaging biomarkers. Specifically, for the ± 20%, ± 30%, and ± 40% response assessment thresholds, 206 (62.8%), 201 (61.3%), and 205 (62.5%) lesions, respectively, had discordant classifications (refer to Table 4). An example of a lesion classified as all response categories is provided in Supplementary Fig. 1. The full lesion classifications for all biomarkers and response assessment thresholds are provided in Supplementary Table 3. Subgroup analysis focusing on specific imaging biomarkers revealed less heterogeneity among the various SUV_peak_ measurements (17.7%, 12.8% and 12.2% of lesions for the ±20%, ±30% and ±40% response assessment thresholds, respectively, Supplementary Table 4) and, excluding only volume, the SUV biomarkers (26.2%, 22.9%, and 23.2%, respectively; refer to Supplementary Table 5), indicating that volume contributes substantially to the observed classification heterogeneity. Out of the 138 patients with identified lesions at baseline, 97 (70.3%), 96 (69.6%), and 98 (71.0%) had at least one lesion with a discordant classification for the response assessment thresholds of ±20%, ±30%, and ±40%, respectively. Conversely, when the biomarker was held constant and the response assessment threshold was changed, the discordance in lesion-level classification ranged from 13.1 to 22.9%, depending on the imaging biomarker (see Table 5).

**Table 4:** Discordance of lesion-level progression classifications between measured quantitative biomarkers at each percentage change threshold.

Response assessment threshold	Classification							Discordant Classification (n, %)
	PR	Stable	Progressing	PR/Stable	Stable/Progressing	PR/Stable/Progressing	PR/Progressing	
± 20%	67	19	36	67	52	36	51	206 (62.8%)
± 30%	53	39	35	77	53	34	37	201 (61.3%)
± 40%	35	59	29	85	68	26	26	205 (62.5%)

PR, Partial response.

**Table 5:** Discordance of lesion-level progression classifications between response assessment thresholds for each imaging biomarker investigated.

Biomarker	Spherical Radius (mm)	Classification					Discordant Classification (n, %)
		PR	Stable	Progressing	PR/Stable	Stable/Progressing	
SUV_peak_ (centered on SUV_max_)	2.50	110	82	65	46	25	71 (21.6%)
	3.75	109	87	62	48	22	70 (21.3%)
	5.00	108	88	61	48	23	71 (21.6%)
	6.25	109	89	59	48	23	71 (21.6%)
	7.50	107	87	59	51	24	75 (22.9%)
	8.75	107	89	62	48	22	70 (21.3%)
SUV_peak_ (highest uptake region)	2.50	110	82	65	46	25	71 (21.6%)
	3.75	109	89	63	46	21	67 (20.4%)
	5.00	108	93	60	43	24	67 (20.4%)
	6.25	108	91	57	45	27	72 (22.0%)
	7.50	108	87	62	48	23	71 (21.6%)
	8.75	104	89	63	52	20	72 (22.0%)
SUV_max_	N/A	110	84	66	44	24	68 (20.7%)
SUV_mean_	N/A	103	86	68	50	21	71 (21.6%)
Volume	N/A	102	75	108	30	13	43 (13.1%)

PR, Partial Response; SUV, Standardised Uptake Value.

### Patterns of progression

**Figure 3:**
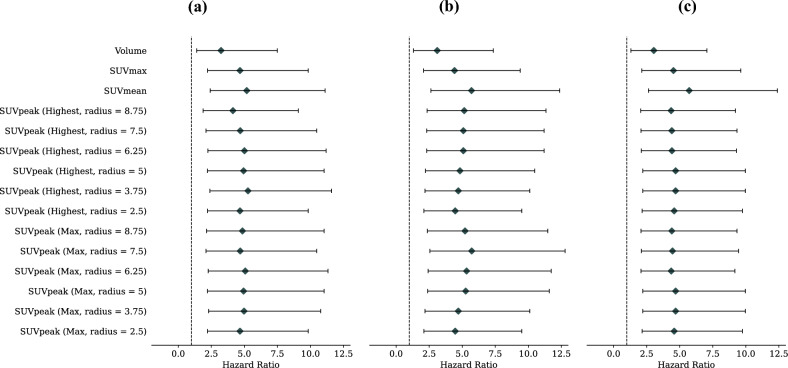
Hazard ratios with 95% confidence intervals of patients classified as having systemic progression relative to patients classified as having no progression for each lesion-level imaging biomarker investigated. Results are presented for: (**a**) ± 20% lesion-level response assessment threshold for progressing and responding lesions, (**b**) ± 30%, and (**c**) ± 40%. In all cases, SUV_mean_ yielded the equal or highest C-Index. Vertical dotted line is plotted at a hazard ratio of 1 for easy visual comparison. All *p* values are less than 0.05, with the exception of volume for all investigated thresholds (after correcting for multiple testing using the Bonferroni method).

For all investigated biomarkers and response assessment thresholds, systemic progressors had a significantly higher risk of death compared to those with no progression (Fig. 3, Supplementary Table 6). After applying the Bonferroni correction to the *p* values for the 45 different hazard ratios calculated, systemic progressors still exhibited a significantly increased risk of death for all imaging biomarkers at each threshold, with the exception of tumour volume (*p* > 0.05 for all response assessment thresholds). Among the biomarkers, SUV_mean_ consistently yielded the largest C-Index when comparing patients with systemic progression to those with no progression. In particular, SUV_mean_ at the ± 30% threshold demonstrated the highest overall C-Index (HR = 5.70, 95% CI = 2.63–12.37, *p* < 0.001, C-Index = 0.69).

**Figure 4:**
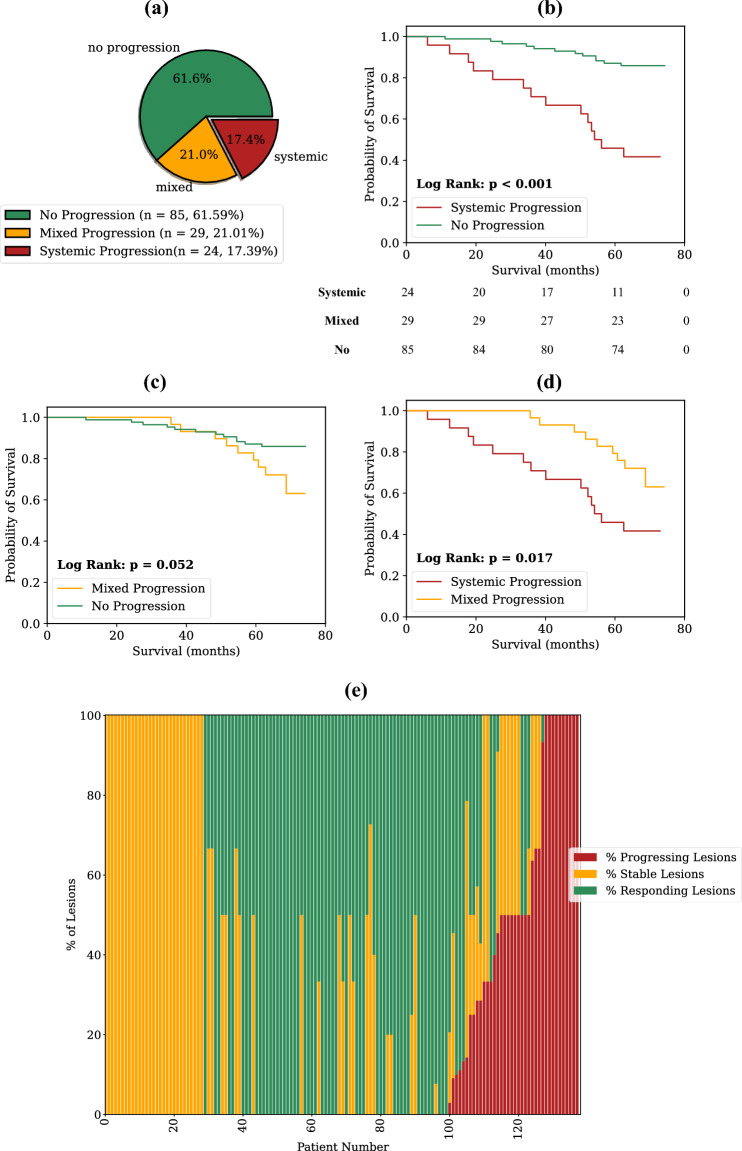
In depth analysis of the properties of the SUV_mean_ biomarker at the ± 30% response assessment threshold. Figure shows: (**a**) pie chart showing percentage of patients with each pattern of progression; Kaplan–Meier curves with annotated log rank *p* values for (**b**) systemic versus no progression, (**c**) mixed versus no progression, (**d**) systemic versus mixed progression, and; (**e**) waterfall plot showing the percentage of each patients' lesions classified in each lesion-level response category (progressing, stable, or responding). Completely responding lesions (i.e., lesions that were not identified on follow-up imaging), are here classified as "responding". The number of patients that are still at risk at a given time point, defined as those patients that have either not experienced death or been censored, are shown below plot (**b**) (time points in the table align with the x-axes of the plot).

**Figure 5:**
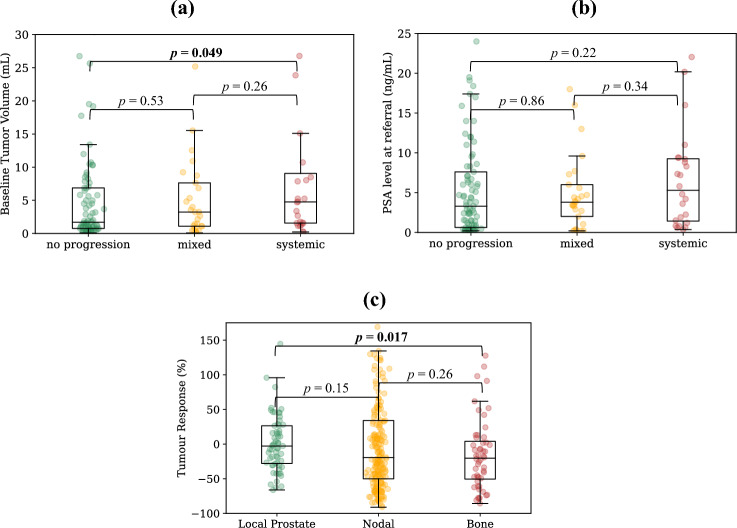
Associations between patterns of progression categories as measured by best performing lesion-level biomarker, SUV_mean_, and (**a**) baseline tumour volume; and (**b**) baseline PSA values. No significant differences were found in tumour response between tumour subgroups of local prostate lesions, nodal lesions and bone lesions as measured by SUV_mean_ (**c**).

Patterns of progression were further defined and analysed for the best performing biomarker (as measured by the C-Index), in this case the SUV_mean_ at the ±30% threshold. Out of the total patient cohort, 85 patients (61.6%) showed no progression, 29 patients (21.0%) had mixed progression, and 24 patients (17.4%) exhibited systemic progression. Kaplan–Meier analysis revealed a statistically significant reduction in survival probability for patients with systemic progression compared to those with no progression (median OS = 54.1 months vs. median OS not reached, log rank *p* < 0.001) and systemic progression versus mixed progression (median OS = 54.1 months vs. median OS not reached, *p* = 0.017, Fig. 4), but not for mixed progression versus no progression (median OS not reached for both, *p* = 0.052). Supplementary Figs. 2 and 3 provide this analysis repeated for SUV_max_ and the PERCIST-recommended biomarker of SUV_peak_ on the highest uptake tumour region with a spherical volume of 1 mL (equating to approximately the radius of 6.25 mm used in this study). Baseline tumour burden was found to be statistically higher in patients with systemic progression relative to those with no progression (median of 4.76 mL vs. 1.69 mL, *p* = 0.049, Fig. 5a). This was not the case, however, for baseline PSA levels between systemic progressors and those with no progression (median of 5.3 ng/mL vs. 3.3 ng/mL, *p* = 0.22, Fig. 5b). There were no statistically significant differences for either the baseline tumour burden or baseline PSA values for all other group combinations (Fig. 5).

Intra-patient lesion response heterogeneity was found to be substantial when analysed for the SUV_mean_ biomarker at the ± 30% threshold. Of the 82 patients that presented with multiple disease sites at baseline imaging, a majority of them (49, 59.8%) had a mixture of responding, stable, and progressing lesions. Considering only the patients treated with systemic hormone therapy, 44% (33/75) had a mixture of classifications at the lesion level. The median tumour response percentage for the 328 matched lesions according to SUV_mean_ was − 14.8% (range: − 91.1–884.9%). Bone lesions had the best response among lesion sub-types (median = − 20.4%), followed by nodal lesions (median = − 19.5%) and local prostate lesions (median = − 2.8%). Only bone and prostate lesions were found to have a significantly different tumour response distribution (*p* = 0.017). However, the differences in tumour response distributions between the other groups were not found to be statistically significant (*p* > 0.05 in pairwise comparison between all groups, Fig. 5c). A majority of the patients overall had at least one responding lesion (88/138, 63.8%), and this remained true when considering only patients with multiple disease sites at baseline (65/82, 79.3%).

### rPPP

**Figure 6:**
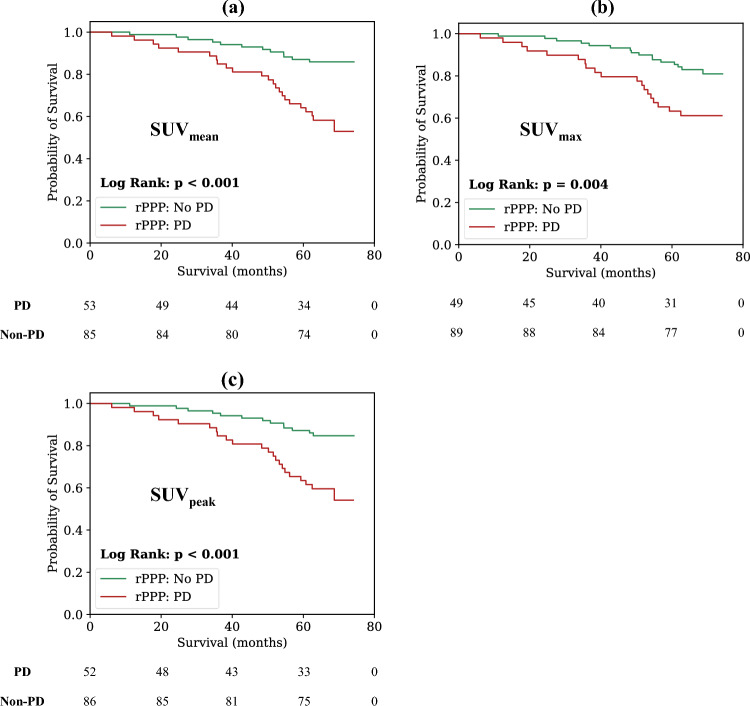
Kaplan–Meier curves showing difference in survival probabilities between patients with PD and those without PD according to the rPPP. Analysis is conducted for: (**a**) SUV_mean_ (**b**) SUV_max_ and (**c**) SUV_peak_. The SUV_peak_ measurement used in this analysis is the PERCIST-recommended one—centered on the highest uptake part of the tumour with a spherical volume of 1 mL. The number of patients that are still at risk at a given time point, defined as those patients that have either not experienced death or been censored, are shown below each plot (time points in the table align with the x-axes of the plot).

The prognostic value of the rPPP criteria in our cohort was also investigated for the best performing biomarker, SUV_mean_, as well as two other commonly used PSMA biomarkers, SUV_max_ and the PERCIST-recommended SUV_peak_. PD as measured by all biomarkers demonstrated a significantly increased risk of death relative to non-PD patients. SUV_mean_ demonstrated the highest increased risk of death (HR = 3.67, 95% CI = 1.82–7.39, *p* < 0.001, C-Index = 0.65), followed by SUV_peak_ (HR = 3.35, 95% CI = 1.69–6.67, *p* < 0.001, C-Index = 0.64) and then SUV_max_ (HR = 2.56, 95% CI = 1.31–4.99, *p* = 0.006, C-Index = 0.62). Kaplan–Meier survival curves for each of these three biomarkers are provided in Fig. 6. Sub-grouping for only the patients receiving anti-androgen treatments, we find that there is still a statistically significant increased risk of death for SUV_mean_ (HR = 4.18, 95% CI = 1.64–10.63, *p* = 0.003, C-Index = 0.67), SUV_peak_ (HR = 3.35, 95% CI = 1.35–8.34, *p* = 0.009, C-Index = 0.65), and SUV_max_ (HR = 3.11, 95% CI = 1.25–7.74, *p* = 0.015, C-Index = 0.64). Further analysis of the rPPP criteria by patient sub-group (initial risk, initial tumour burden, treatment type) is presented in Supplementary Table 7.

## Discussion

PSMA-targeted PET imaging is increasingly being used in response assessment settings for PCa patients, but the evidence of its prognostic utility in early disease BCR PCa patients is currently limited. One of the important advantages that whole-body molecular imaging confers to clinicians is the ability to assess progression at individual disease sites, which contrasts with biochemical progression measurements in the form of PSA, which have no ability to detect heterogeneous response across different disease sites^28^. In this work, we comprehensively assess progression at the lesion level according to a range of different imaging biomarkers at different response thresholds, and evaluate patient-level progression criteria derived from these lesion-level measurements. The results have important clinical implications, and add crucial evidence for the prognostic value of PSMA PET quantitation in the response assessment setting for BCR PCa patients.

When assessing radiographic progression at the lesion level in PCa, the choice of imaging biomarker can drastically impact the resulting classification of that disease site. Quantification of burden at the lesion-level can be undertaken using any number of different biomarkers, including SUV_max_, SUV_mean_, SUV_peak_ (as recommended by the PERCIST criteria) and volume (which is implicitly incorporated into patient level frameworks such as RECIP 1.0, which requires a whole-body measurement of PSMA-positive tumour volume), among many others^15,20,24,29^. Our study found substantial heterogeneity in lesion-level progression classifications (progressing, stable, and responding) dependent on the imaging biomarker used for assessing response (> 60% of lesions with discordant classifications). This has important clinical implications—radiographic assessment of lesion-level response can yield significantly different interpretations depending on the imaging biomarker chosen for the task. Indeed, at all response assessment thresholds investigated, some lesions were variably classified as the full spectrum of possibilities (Partial Response/Stable/Progressing) depending on the biomarker chosen. This could have a profound influence on the resulting clinical interpretation of the success of therapeutic intervention, especially in low disease burden patients with limited metastatic sites, where a lesion can be classified as either “progressing” or “responding” simply by changing the imaging biomarker used for the assessment (see Supplementary Fig. 1 for an example of this).

Given the considerable heterogeneity in lesion-level classifications amongst imaging biomarkers, an important question arises—which biomarker (or biomarkers) should be used? This question is complex and dependent on numerous factors, such as ease of use in clinical practice, correlation with relevant clinical endpoints, and reproducibility of the biomarker among operators. This work does not attempt to definitively answer this question—rather, we take a step towards answering a part of this question by assessing the correlation between patient-level patterns of progression derived from lesion-level biomarker measurements and the important clinical endpoint of patient OS. To assess the prognostic ability of the biomarkers at each threshold, we characterised the progression dynamics according to pre-defined patterns of progression. Overall, regardless of the response assessment threshold chosen, the SUV_mean_ had the equal or highest C-index for predicting decreased survival in patients with systemic progression compared to those without progression. The ±30% threshold performed the best, with an HR of 5.70 (95% CI = 2.63–12.37, *p* < 0.001) and a C-Index of 0.69. These findings suggest that SUV_mean_ is a valuable biomarker for assessing lesion-level progression. However, it is important to note that all other biomarkers tested (except for volume after adjusting for multiple testing) also demonstrated significant increases in the risk of death for systemic progressors relative to patients without progression. Thus, despite the substantial heterogeneity found in lesion-level classifications, most biomarkers retain prognostic significance when these lesion-level classifications are packaged into patterns of progression, and considering other factors, this may lead to an alternative choice of biomarker for use depending on the clinical context. For instance, SUV_mean_, requires full delineations of each metastatic site to calculate, and while semi-automated and automated AI-based solutions exist for segmenting metastatic disease from PSMA PET scans^30–32^, not all centres around the world will have access to these technologies. This could make such an approach infeasible for patients with high disease burden. SUV_max_ could be used as a more practical alternative in high disease burden cases, where the maximum voxel in a high uptake area can be localised without precise tumour delineations^33^. SUV_max_ quantification in prostate lesions has demonstrated high inter-reader reproducibility, however, the use of the single highest voxel value in a region for tumour response quantification can be adversely affected by noise in the acquired image^34,35^. An alternative to SUV_max_ is the PERCIST-recommended biomarker, SUV_peak_, which is more robust to noise due to volume averaging. However, response quantification using SUV_peak_ is dependent on both the size of the spherical volume defining the region of interest and where that sphere is placed, as demonstrated not only in this study, but also an investigation by Vanderhoek et al.^25^. There is also the possibility that the specified region of interest over which the SUV_peak_ is quantified might include voxels outside of the tumour volume. It’s worth noting also that due to the limited spatial resolution of PET imaging, partial volume effects can affect biomarker quantitation accuracy^36^. This leads to an underestimation of lesion activity because of activity dilution at the borders of the region of interest from volume averaging, and makes the lesion appear larger than it is in reality.

A closer look at the patterns of progression for the SUV_mean_ biomarker at the ±30% threshold illuminates more clinically relevant conclusions. Patients with systemic, mixed and no progression all had different survival outcomes, with systemic progressors demonstrating statistically lower survival probabilities than both patients with mixed (*p* = 0.017) and no progression (*p* < 0.001). This accords with the results of a study by Osorio et al.^21^, that analysed lesion-level patterns of progression for two cohorts of patients, one with non-small cell lung cancer (NSCLC) and one with metastatic carcinoma with mismatch repair deficiency (MMRD) who were treated with programmed cell death protein (PD-1) blockade therapies. In both cohorts examined, mixed progressors were found to have improved OS relative to systemic progressors, although not to statistical significance in the MMRD cohort (*p* = 0.07). Notably, our analysis used the same definitions of progression patterns as this study, suggesting that they retain their prognostic significance across a range of metastatic cancer types and can be applied to PCa at the stage of BCR. Intra-patient heterogeneity of lesion-level response was also very common in patients with multiple disease sites, with a majority of them (59.8%) having a mixture of progressing, stable and responding lesions between imaging assessments. 44% of patients who underwent systemic hormone therapy also presented with a mixture of classifications at the lesion level. This implies underlying biological heterogeneity between metastatic disease sites, of which previous studies have shown evidence for in PCa as well as other cancer types such as melanoma, NSCLC and gastric cancer^19,20^. Furthermore, a majority of patients in this study demonstrated response in at least one metastatic disease site. This finding highlights the potential importance of therapy continuation in cases where certain disease sites are responding favourably, even if other sites show limited response or progression. Such valuable information can only be captured with a lesion-level assessment of disease progression. It emphasizes the significance of incorporating such analyses in future studies and clinical trials to enhance our understanding of treatment responses and guide clinical decision-making.

This work also confirms the prognostic value of a radiographic interpretation of the PPP criteria (rPPP) for patient progression in the BCR setting using SUV_mean_, SUV_max_ and the PERCIST-recommended SUV_peak_. The rPPP response framework validated in this work is consistent with the PSMA PET/CT response assessment consensus statement published by Fanti et al*.*^27^, where the presence of one new lesion in early recurrent PCa is enough to be considered PD. This work also adds evidence that confirms the value of the recently published PROMISE v2 framework which recommends the PPP be used for response assessment in PCa patients with limited metastatic disease, and we show that the assessment can be done purely radiographically while still maintaining prognostic value^37^. Furthermore, the prognostic value of the rPPP is maintained even when sub-grouping for just the patients that received anti-hormone therapy between scans. This sub-group analysis was conducted because of the growing evidence that androgen blockade can increase PSMA expression and affect PSMA PET quantitation^38,39^, potentially resulting in the conflation of true progression with receptor up-regulation in response to anti-hormone therapy. We acknowledge that this work does not account for this phenomenon, but note that the maintenance of prognostic value in the sub-group analysis is promising evidence that the rPPP framework can be used in the BCR setting even in such populations. Further subgroup analysis by treatment type also demonstrated that the rPPP remains highly prognostic for all treatment sub-groups for these three biomarkers with the exception of patients who underwent no therapy, suggesting that disease progression despite treatment is associated with a much poorer patient prognosis than progression when no treatment is administered. This conclusion should be tempered, however, by the relatively small number of patients in the no treatment sub-group (n = 39). Future prospective clinical trials with standardised treatment regimens are necessary to confirm these results.

This study has some limitations that should be noted. The retrospective nature of this work means that the findings warrant further prospective validation. It would be infeasible for such prospective clinical trials to evaluate all biomarkers—rather, the results of this work in combination with other pertinent considerations mentioned above can be used to narrow down a small number of candidate biomarkers. Such studies should also ideally incorporate delineations from more than one nuclear medicine physician if possible, as this was a limitation of the present work. Additionally, the patient cohort overall had relatively low disease burden PCa (median 2 lesions per patient) Therefore, the conclusions drawn from this study are applicable only to this cohort and may not extend to patients with more extensive disease burden. Further research is required to validate the prognostic utility of lesion-level analysis with respect to patient OS in more advanced disease populations. Indeed, it is possible that the patterns of progression definitions used in this work may need to be refined for patients with high disease burden. One can imagine a scenario, for example, where a patient with considerably advanced disease presenting with potentially ~ 100 disease sites is classified as having “systemic progression” in response to ^177^Lu-PSMA therapy because a very small minority of those sites (say three) are unresponsive to therapy and increasing in uptake, even when the remainder may respond well. Response criteria incorporating overall tumour burden measurements, such as RECIP 1.0, might be more informative in such cases, as recommended by the PROMISE v2 framework^37^. Lastly, the patient cohort in this work were not administered a standardised treatment regimen, which limits the conclusions that can be drawn for individual treatment modalities. The overall conclusions about the prognostic utility of these patterns of progression and the rPPP will thus need to be verified for individual treatment modalities in future works, which would ideally be prospective. It is also possible that subsequent treatments administered to patients after the follow-up scan could confound the survival analysis conducted, and this is something that future works investigating the prognostic power of imaging metrics in this early disease setting should investigate.

In conclusion, evaluating radiographic response from ^68^Ga-PSMA PET images in metastatic PCa at the lesion level is subject to significant heterogeneity among imaging biomarkers, which could have important implications for assessing therapy response at individual metastatic sites. However, despite this heterogeneity in lesion-level classifications, almost all biomarkers remained prognostically significant when patient-level patterns of progression were defined from these lesion-level classifications, with our results suggesting SUV_mean_ is the most prognostically significant. The three progression groups had distinct survival outcomes, with systemic progressors at the highest risk of death, demonstrating the prognostic significance of lesion-level assessments in PSMA PET in the BCR setting. rPPP was also shown to be prognostic in this cohort, demonstrating that response assessment using PSMA PET is prognostically relevant even at early stages of metastatic PCa. Future prospective clinical trials should aim to validate the promising findings of this work.

### Supplementary Information

Supplementary Figure S1.

Supplementary Figure S2.

Supplementary Figure S3.

Supplementary Tables.

## Data Availability

The datasets generated during and/or analyzed during the current study are not publicly available due to privacy and ethics restrictions. The data that support the findings of this study are available from the corresponding author upon reasonable request, but no person-identifying data can be provided.
