# Supplementary material for: Quantitative [68Ga]Ga-PSMA-11 PET biomarkers for the analysis of lesion-level progression in biochemically recurrent prostate cancer: a multicentre study
Source: Sci Rep. 2023 Oct 17;13:17673. doi: 10.1038/s41598-023-45106-2 (PMC10582101; doi:10.1038/s41598-023-45106-2)
Supplement: Supplementary file 1 — Supplementary Figure S1. [file 41598_2023_45106_MOESM1_ESM.pptx]

## Slide 1
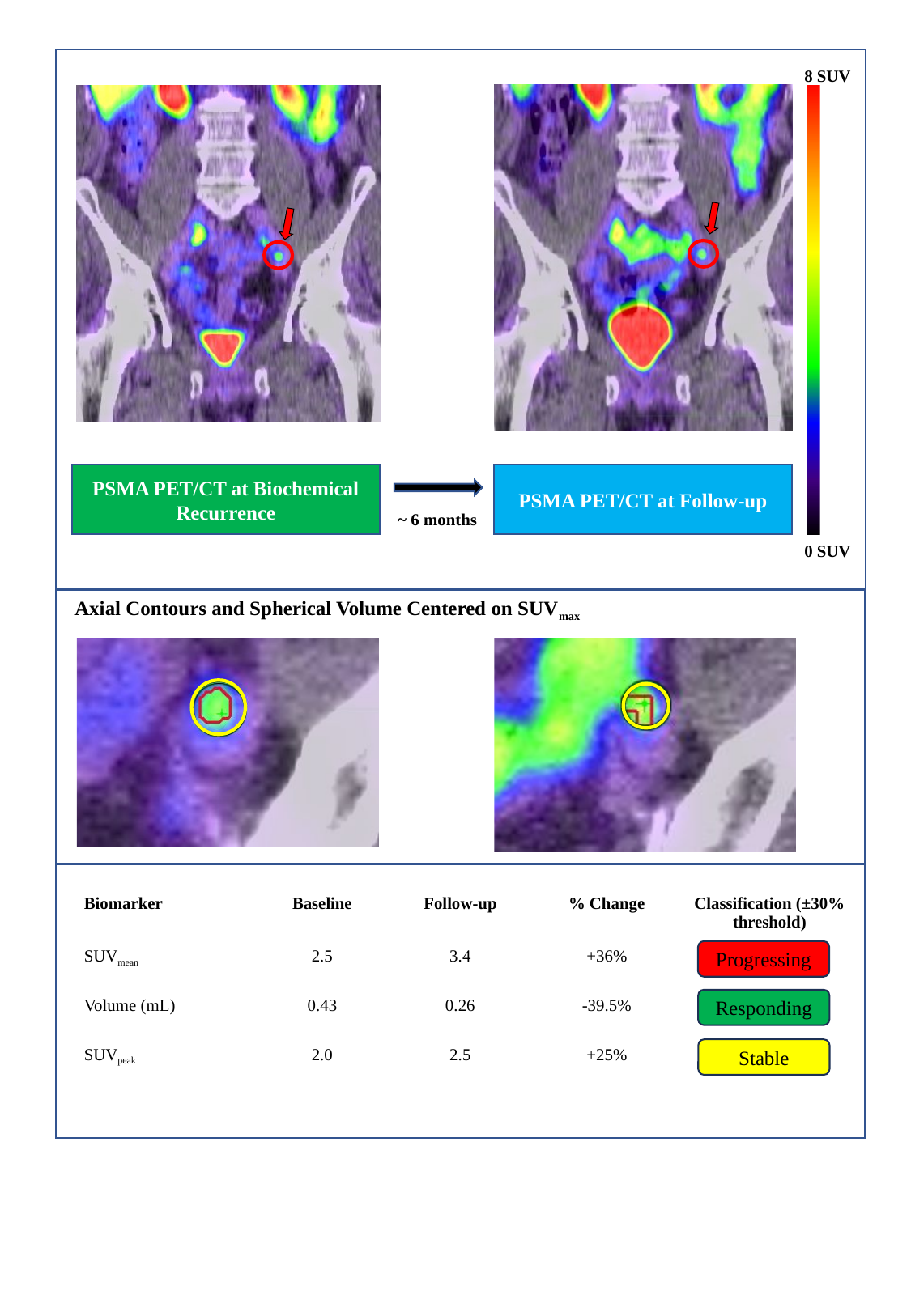

8 SUV
15
PSMA PET/CT at Biochemical Recurrence
PSMA PET/CT at Follow-up
~ 6 months
0 SUV
Axial Contours and Spherical Volume Centered on SUVmax
| Biomarker | Baseline | Follow-up | % Change | Classification (±30% threshold) |
| --- | --- | --- | --- | --- |
| SUVmean | 2.5 | 3.4 | +36% | |
| Volume (mL) | 0.43 | 0.26 | -39.5% | |
| SUVpeak | 2.0 | 2.5 | +25% | |
Progressing
Responding
Stable
0

## Slide 2
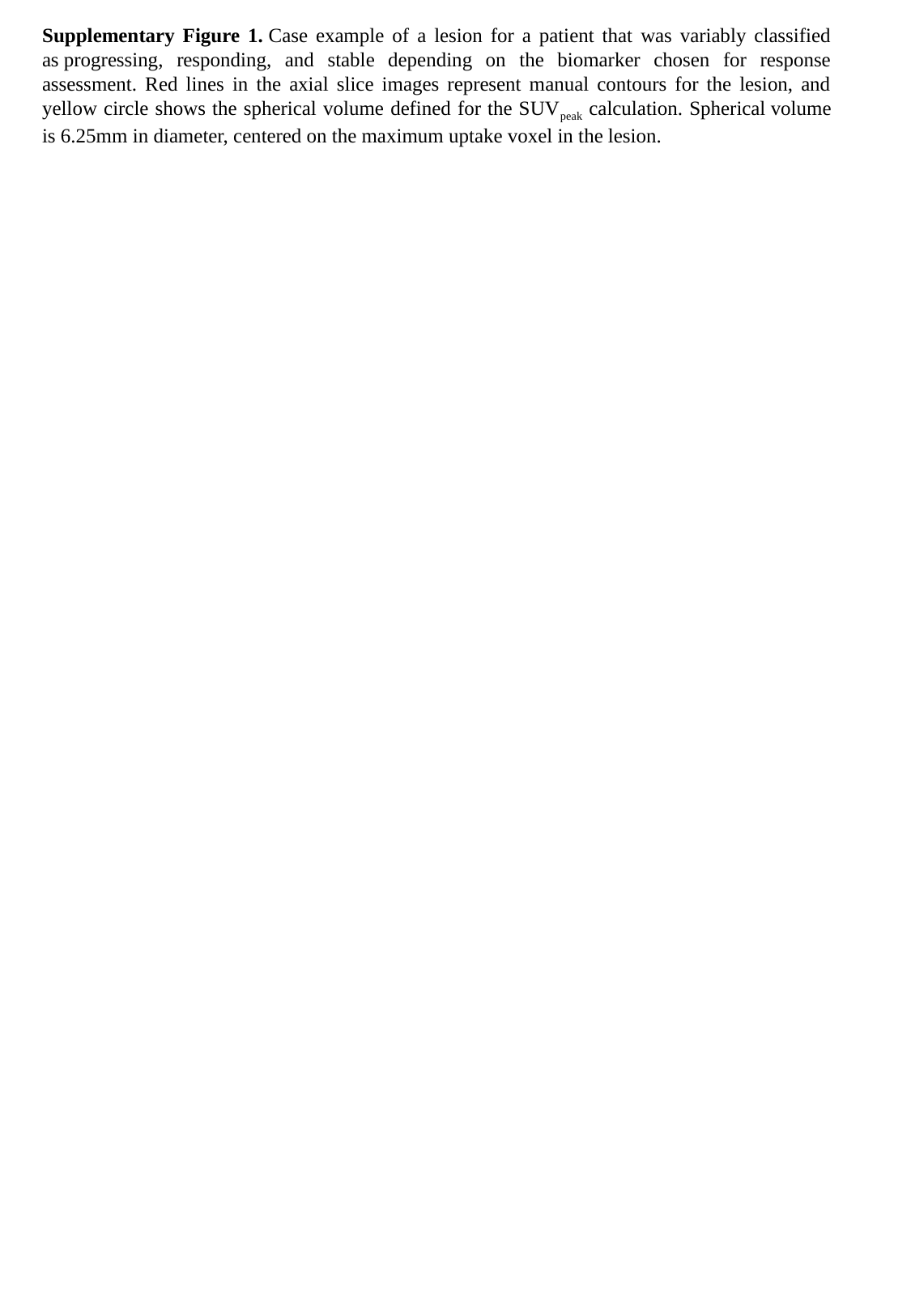

Supplementary Figure 1. Case example of a lesion for a patient that was variably classified as progressing, responding, and stable depending on the biomarker chosen for response assessment. Red lines in the axial slice images represent manual contours for the lesion, and yellow circle shows the spherical volume defined for the SUVpeak calculation. Spherical volume is 6.25mm in diameter, centered on the maximum uptake voxel in the lesion.
