# Supplementary material for: Quantitative [68Ga]Ga-PSMA-11 PET biomarkers for the analysis of lesion-level progression in biochemically recurrent prostate cancer: a multicentre study
Source: Sci Rep. 2023 Oct 17;13:17673. doi: 10.1038/s41598-023-45106-2 (PMC10582101; doi:10.1038/s41598-023-45106-2)
Supplement: Supplementary file 2 — Supplementary Figure S2. [file 41598_2023_45106_MOESM2_ESM.pptx]

## Slide 1
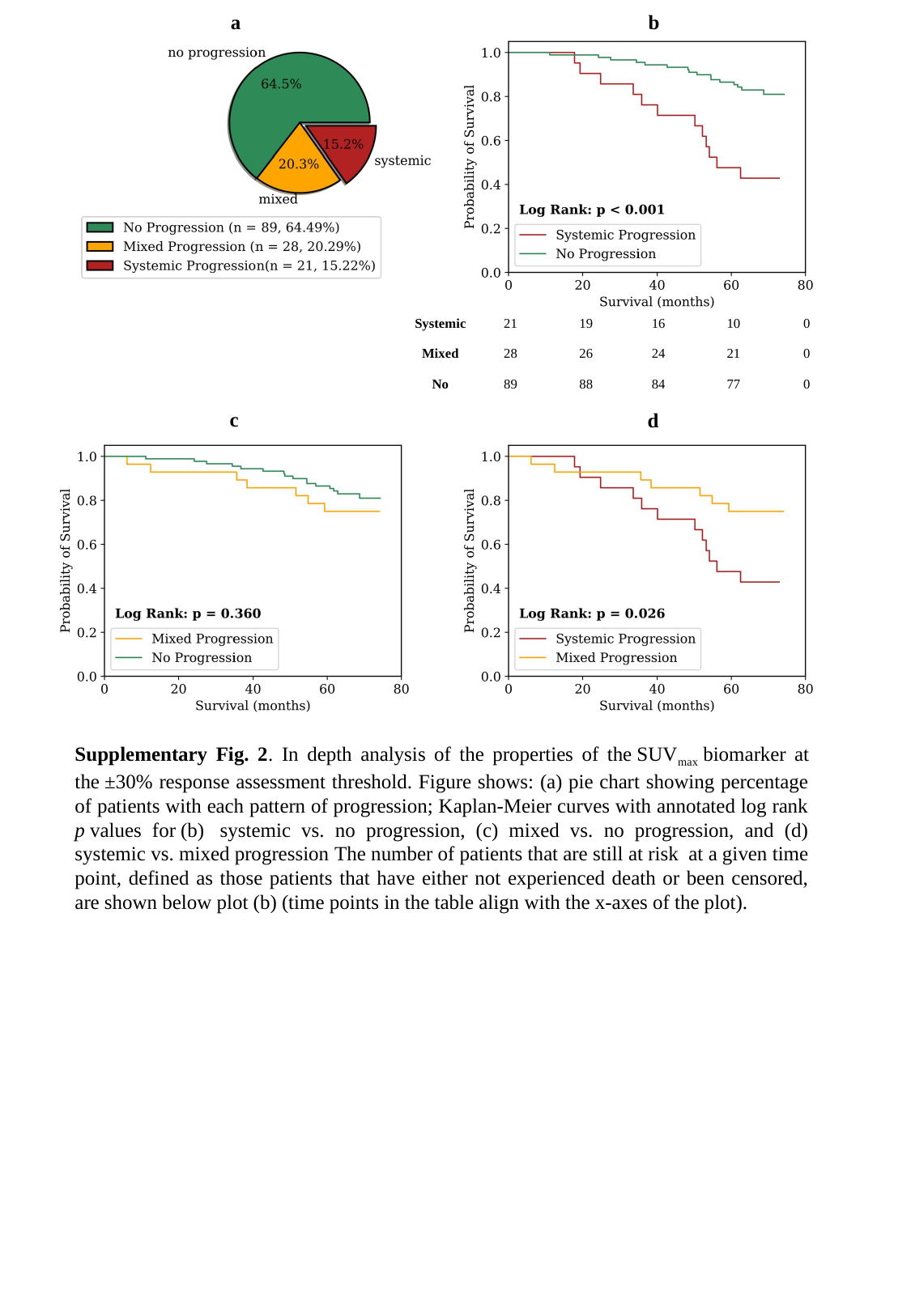

a
b
| Systemic | 21 | 19 | 16 | 10 | 0 |
| --- | --- | --- | --- | --- | --- |
| Mixed | 28 | 26 | 24 | 21 | 0 |
| No | 89 | 88 | 84 | 77 | 0 |
c
d
Supplementary Fig. 2. In depth analysis of the properties of the SUVmax biomarker at the ±30% response assessment threshold. Figure shows: (a) pie chart showing percentage of patients with each pattern of progression; Kaplan-Meier curves with annotated log rank p values for (b)  systemic vs. no progression, (c) mixed vs. no progression, and (d) systemic vs. mixed progression The number of patients that are still at risk  at a given time point, defined as those patients that have either not experienced death or been censored, are shown below plot (b) (time points in the table align with the x-axes of the plot).
0
