# Supplementary material for: Quantitative [68Ga]Ga-PSMA-11 PET biomarkers for the analysis of lesion-level progression in biochemically recurrent prostate cancer: a multicentre study
Source: Sci Rep. 2023 Oct 17;13:17673. doi: 10.1038/s41598-023-45106-2 (PMC10582101; doi:10.1038/s41598-023-45106-2)
Supplement: Supplementary file 4 — Supplementary Tables. [file 41598_2023_45106_MOESM4_ESM.docx]

Supplementary Table 1: Summary of PET scanner parameters for patients imaged at each institution.

| **Imaging Centre** | **Scanner** | **Reconstruction Method** | **Pixel Spacing (mm^2^)** |
| --- | --- | --- | --- |
| SCGH | Siemens Biograph 64 | PSF + Time of Flight (2i 21s) | 4.07 × 4.07 |
| FSH | Siemens Biograph 128 | OSEM + Time of Flight (2i 21s) | 4.07 × 4.07 |

Supplementary Table 2: Description of the biomarkers calculated in this study, all of which were calculated from the patient PET image.

| **Biomarker** | **Description** |
| --- | --- |
| SUV_max_ | SUV value of the voxel with the highest uptake in the defined contour of the lesion. |
| SUV_mean_ | Mean SUV of all voxels included in the lesion contour. |
| SUV_peak_ | Mean SUV of all voxels included in a defined spherical region of interest. Spherical region of interest is defined by two factors:   - Radius (in mm) of the sphere. - Location of the sphere, being centered either on the SUV_max_ of the contoured lesion, or iterated over all voxels in the lesion contour to find the highest uptake area. |
| Volume | Number of voxels included in the defined contour of the lesion multiplied by the volume of a single voxel. |

Abbreviations: SUV – Standardised Uptake Value

Supplementary Table 3: Full response classifications for all matched lesions between baseline and follow-up imaging.

| **Biomarker** | **Spherical Radius (mm)** | **±20%** | | | **±30%** | | | **±40%** | | |
| --- | --- | --- | --- | --- | --- | --- | --- | --- | --- | --- |
|  |  | **PR** | **Stable** | **PD** | **PR** | **Stable** | **PD** | **PR** | **Stable** | **PD** |
| **SUV_peak_ (Centered on SUV_max_)** |  |  |  |  |  |  |  |  |  |  |
|  | 2.50 | 156 | 82 | 90 | 131 | 127 | 70 | 110 | 153 | 65 |
|  | 3.75 | 157 | 87 | 84 | 131 | 125 | 72 | 109 | 157 | 62 |
|  | 5.00 | 156 | 88 | 84 | 130 | 126 | 72 | 108 | 159 | 61 |
|  | 6.25 | 157 | 89 | 82 | 132 | 126 | 70 | 109 | 160 | 59 |
|  | 7.50 | 158 | 87 | 83 | 132 | 125 | 71 | 107 | 162 | 59 |
|  | 8.75 | 155 | 89 | 84 | 130 | 127 | 71 | 107 | 159 | 62 |
| **SUV_peak_ (Highest Uptake region)** |  |  |  |  |  |  |  |  |  |  |
|  | 2.50 | 156 | 82 | 90 | 131 | 127 | 70 | 110 | 153 | 65 |
|  | 3.75 | 155 | 89 | 84 | 131 | 125 | 72 | 109 | 156 | 63 |
|  | 5.00 | 151 | 93 | 84 | 131 | 125 | 72 | 108 | 160 | 60 |
|  | 6.25 | 153 | 91 | 84 | 133 | 122 | 73 | 108 | 163 | 57 |
|  | 7.50 | 156 | 87 | 85 | 130 | 125 | 73 | 108 | 158 | 62 |
|  | 8.75 | 156 | 89 | 83 | 124 | 132 | 72 | 104 | 161 | 63 |
| **SUV_max_** | N/A | 154 | 84 | 90 | 133 | 124 | 71 | 110 | 152 | 66 |
| **SUV_mean_** | N/A | 153 | 86 | 89 | 126 | 124 | 78 | 103 | 157 | 68 |
| **Volume** | N/A | 132 | 75 | 121 | 119 | 94 | 115 | 102 | 118 | 108 |

Supplementary Table 4: Discordance of lesion-level progression classifications including only the different types of SUV_peak_ measurements at each percentage change threshold.

| **Response Assessment Threshold** | **Classification** | | | | | | **Discordant Classification (n, %)** |
| --- | --- | --- | --- | --- | --- | --- | --- |
|  | **Partial Response** | **Stable** | **Progressing** | **Partial Response / Stable** | **Stable / Progressing** | **Partial Response / Stable / Progressing** |  |
| **±20%** | 139 | 57 | 74 | 32 | 24 | 2 | 58 (17.7%) |
| **±30%** | 117 | 108 | 61 | 23 | 19 | 0 | 42 (12.8%) |
| **±40%** | 97 | 136 | 55 | 22 | 18 | 0 | 40 (12.2%) |

Supplementary Table 5: Discordance of lesion-level progression classifications including only SUV measurements (no volume) at each percentage change threshold.

| **Response Assessment Threshold** | **Classification** | | | | | | **Discordant Classification (n, %)** |
| --- | --- | --- | --- | --- | --- | --- | --- |
|  | **Partial Response** | **Stable** | **Progressing** | **Partial Response / Stable** | **Stable / Progressing** | **Partial Response / Stable / Progressing** |  |
| **±20%** | 130 | 45 | 67 | 41 | 37 | 8 | 86 (26.2%) |
| **±30%** | 108 | 87 | 58 | 39 | 33 | 3 | 75 (22.9%) |
| **±40%** | 81 | 119 | 52 | 46 | 29 | 1 | 76 (23.2%) |

Supplementary Table 6: Hazard ratios, *p* values and C-Indices calculated for each biomarker at each response assessment threshold using univariate Cox regression analysis. Values reflect the comparison between systemic progressors and patients with no progression.

| **Biomarker** | **±20%** | | | **±30%** | | | **±40%** | | |
| --- | --- | --- | --- | --- | --- | --- | --- | --- | --- |
|  | **HR [95% CI]** | ***p^#^*** | **C-Index** | **HR [95% CI]** | ***p^#^*** | **C-Index** | **HR [95% CI]** | ***p^#^*** | **C-Index** |
| **SUV_peak_ (Centered on SUV_max_)** |  |  |  |  |  |  |  |  |  |
| 2.50 mm | 4.67  [2.22 - 9.83] | 0.002 | 0.67 | 4.47  [2.10 - 9.50] | 0.004 | 0.65 | 4.58  [2.16 - 9.74] | 0.003 | 0.65 |
| 3.75 mm | 4.98  [2.30 - 10.78] | 0.002 | 0.67 | 4.70  [2.19 - 10.11] | 0.003 | 0.66 | 4.69  [2.21 - 9.97] | 0.003 | 0.65 |
| 5.00 mm | 4.94  [2.21 - 11.03] | 0.004 | 0.67 | 5.26  [2.39 - 11.59] | 0.002 | 0.67 | 4.69  [2.21 - 9.97] | 0.003 | 0.65 |
| 6.25 mm | 5.07  [2.27 - 11.33] | 0.003 | 0.67 | 5.33  [2.42 - 11.73] | 0.001 | 0.67 | 4.36  [2.07 - 9.17] | 0.005 | 0.64 |
| 7.50 mm | 4.69  [2.10 - 10.47] | 0.007 | 0.66 | 5.72  [2.56 - 12.78] | < 0.001 | 0.68 | 4.45  [2.10 - 9.45] | 0.004 | 0.64 |
| 8.75 mm | 4.85  [2.14 - 11.03] | 0.007 | 0.67 | 5.21  [2.37 - 11.47] | 0.002 | 0.67 | 4.40  [2.08 - 9.33] | 0.005 | 0.64 |
| **SUV_peak_ (Highest Uptake region)** |  |  |  |  |  |  |  |  |  |
| 2.50 mm | 4.67  [2.22 - 9.83] | 0.002 | 0.67 | 4.47  [2.10 - 9.50] | 0.004 | 0.65 | 4.58  [2.16 - 9.74] | 0.003 | 0.65 |
| 3.75 mm | **5.27**  **[2.40 - 11.60]** | **0.002** | **0.68** | 4.70  [2.19 - 10.11] | 0.003 | 0.66 | 4.69  [2.21 - 9.97] | 0.003 | 0.65 |
| 5.00 mm | 4.94  [2.21 - 11.03] | 0.004 | 0.67 | 4.83  [2.22 - 10.48] | 0.003 | 0.66 | 4.69  [2.21 - 9.97] | 0.003 | 0.65 |
| 6.25 mm | 5.00  [2.24 - 11.18] | 0.004 | 0.67 | 5.09  [2.31 - 11.19] | 0.002 | 0.67 | 4.41  [2.10 - 9.28] | 0.004 | 0.64 |
| 7.50 mm | 4.69  [2.10 - 10.47] | 0.007 | 0.66 | 5.09  [2.31 - 11.19] | 0.002 | 0.67 | 4.40  [2.08 - 9.33] | 0.005 | 0.64 |
| 8.75 mm | 4.13  [1.88 - 9.08] | 0.018 | 0.65 | 5.15  [2.34 - 11.33] | 0.002 | 0.67 | 4.35  [2.05 - 9.22] | 0.006 | 0.64 |
| **SUV_max_** | 4.67  [2.22 - 9.83] | 0.002 | 0.67 | 4.41  [2.08 - 9.38] | 0.005 | 0.65 | 4.53  [2.13 - 9.62] | 0.004 | 0.65 |
| **SUV_mean_** | **5.18**  **[2.42 - 11.11]** | **0.001** | **0.68** | **5.70**  **[2.63 – 12.37]** | **< 0.001** | **0.69** | **5.72**  **[2.64 - 12.38]** | **< 0.001** | **0.68** |
| **Volume** | 3.24  [1.40 - 7.49] | 0.274 | 0.64 | 3.11  [1.32 - 7.35] | 0.436 | 0.63 | 3.04  [1.31 - 7.06] | 0.435 | 0.62 |

^#^ Bonferroni correction applied.

Supplementary Table 7: Prognostic value of the rPPP in a patient sub-group analysis. Hazard ratios reflect increased risk of death for patients classified with progressive disease relative to those without progressive disease.

| **Sub-group analysis** | **SUV_mean_** | | | **SUV_max_** | | | **SUV_peak_ (PERCIST recommended)** | | |
| --- | --- | --- | --- | --- | --- | --- | --- | --- | --- |
|  | **HR [95% CI]** | ***p*** | **C-Index** | **HR [95% CI]** | ***p*** | **C-Index** | **HR [95% CI]** | ***p*** | **C-Index** |
| **Treatment type** |  |  |  |  |  |  |  |  |  |
| Any Treatment  (n = 99) | 4.31  [1.80 - 10.28] | 0.001 | 0.67 | 3.36  [1.43 - 7.87] | 0.005 | 0.65 | 3.36  [1.43 - 7.87] | 0.005 | 0.65 |
| Any Systemic treatment  (n = 76) | 3.97  [1.56 – 10.09] | 0.004 | 0.66 | 2.96  [1.19 – 7.37] | 0.020 | 0.64 | 3.18  [1.28 - 7.92] | 0.013 | 0.64 |
| Any ADT  (n = 75) | 4.18  [1.64 - 10.63] | 0.003 | 0.67 | 3.11  [1.25 - 7.74] | 0.015 | 0.64 | 3.35  [1.35 – 8.34] | 0.009 | 0.65 |
| No treatment  (n = 39) | 2.29  [0.70 – 7.47] | 0.168 | 0.60 | 1.47  [0.49 – 4.38] | 0.491 | 0.57 | 2.87  [0.88 – 9.32] | 0.080 | 0.63 |
| **Initial risk** |  |  |  |  |  |  |  |  |  |
| Low / Intermediate (n = 67) | 3.62  [1.40 – 9.36] | 0.008 | 0.65 | 2.46  [0.97 – 6.25] | 0.058 | 0.61 | 3.13  [1.23 – 7.95] | 0.016 | 0.64 |
| High  (n =68) | 3.34  [1.16 – 9.62] | 0.026 | 0.64 | 2.56  [0.93 – 7.06] | 0.070 | 0.63 | 3.38  [1.17 – 9.76] | 0.024 | 0.64 |
| **Baseline Tumour Burden** |  |  |  |  |  |  |  |  |  |
| < Median  (n = 69) | 4.08  [1.19 - 13.97] | 0.025 | 0.67 | 4.08  [1.19 - 13.97] | 0.025 | 0.67 | 3.79  [1.11 – 12.97] | 0.034 | 0.66 |
| ≥ Median  (n = 69) | 3.08  [1.31 – 7.23] | 0.010 | 0.63 | 1.94  [0.87 - 4.34] | 0.105 | 0.59 | 2.95  [1.28 - 6.76] | 0.010 | 0.63 |
